# BM-IQE: An Image Quality Evaluator with Block-Matching for Both Real-Life Scenes and Remote Sensing Scenes

**DOI:** 10.3390/s20123472

**Published:** 2020-06-19

**Authors:** Ningshan Xu, Dongao Ma, Guoqiang Ren, Yongmei Huang

**Affiliations:** 1 Institute of Optics and Electronics, Chinese Academy of Sciences, Chengdu 610209, China; xuningshan16@mails.ucas.ac.cn (N.X.); huangym@ioe.ac.cn (Y.H.); 2 The School of Electronic, Electrical and Communication Engineering, University of Chinese Academy of Sciences, Beijing 100049, China; 3 Aerospace Information Research Institute, Chinese Academy of Sciences, Beijing 100101, China; mada@radi.ac.cn

**Keywords:** imaging performance, blind image quality assessment, block-matching, remote sensing

## Abstract

Like natural images, remote sensing scene images; of which the quality represents the imaging performance of the remote sensor, also suffer from the degradation caused by imaging system. However, current methods measuring the imaging performance in engineering applications require for particular image patterns and lack generality. Therefore, a more universal approach is demanded to assess the imaging performance of remote sensor without constraints of land cover. Due to the fact that existing general-purpose blind image quality assessment (BIQA) methods cannot obtain satisfying results on remote sensing scene images; in this work, we propose a BIQA model of improved performance for natural images as well as remote sensing scene images namely BM-IQE. We employ a novel block-matching strategy called Structural Similarity Block-Matching (SSIM-BM) to match and group similar image patches. In this way, the potential local information among different patches can get expressed; thus, the validity of natural scene statistics (NSS) feature modeling is enhanced. At the same time, we introduce several features to better characterize and express remote sensing images. The NSS features are extracted from each group and the feature vectors are then fitted to a multivariate Gaussian (MVG) model. This MVG model is therefore used against a reference MVG model learned from a corpus of high-quality natural images to produce a basic quality estimation of each patch (centroid of each group). The further quality estimation of each patch is obtained by weighting averaging of its similar patches’ basic quality estimations. The overall quality score of the test image is then computed through average pooling of the patch estimations. Extensive experiments demonstrate that the proposed BM-IQE method can not only outperforms other BIQA methods on remote sensing scene image datasets but also achieve competitive performance on general-purpose natural image datasets as compared to existing state-of-the-art FR/NR-IQA methods.

## 1. Introduction

**Figure 1:**
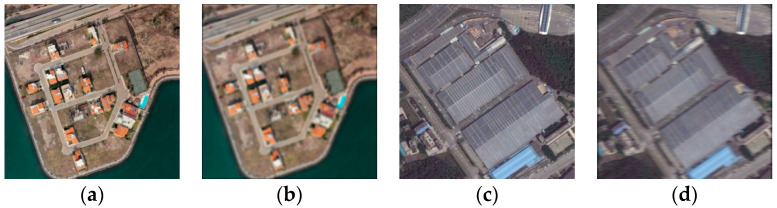
Examples of simulated degraded remote sensing images. (**a**,**c**) are pristine images. (**b**,**d**) are distorted by out-of-focus and platform flutter distortions, respectively.

Real-time monitoring the performance of imaging equipment is important in practical applications such as environmental monitoring and resources exploration [1,2]. In particular, the imaging performance of on-orbit space remote sensors can only be assessed via processing and analyzing the images transmitted from the satellite. Like natural images, remote sensing images also suffer from the degradations caused by the imaging system (as shown in Figure 1). The most salient and primary impact of image degradation is the decrease of image definition, that is, the decrease of visual perception effect which influences the subsequent remote sensing image interpretation. Therefore, remote sensing image quality assessment (RS-IQA) becomes helpful. Current solutions for evaluating the performance of remote sensors include the Target method [3], the Knife-edge method [4], the Pulse method [5], and others. However, in the Target method, not all on-orbit space remote sensors can obtain target images; and in the Knife-edge method and the Pulse method, it is challenging to provide every image returned with effective Knife-edges or pulses. Except for harsh imaging conditions, such deficiencies cause these methods to not be feasible or generalizable to other types of remote sensors. At present, it lacks normative approaches to assess the imaging performance of remote sensors. Thus, it is urgent to develop a universal method to evaluate the imaging performance of remote sensors via RS-IQA.

### 1.1. Related Works

Current approaches to assess the performance of remote sensors typically include those in references [3,4,5]. To the best of our knowledge, there is no IQA method dedicated to RS images, and thus, the development of IQA methods for natural images is reported. In general, IQA methods can be categorized into two parts—subjective assessment methods and objective assessment methods. Due to the fact that the subjective way of monitoring the image quality by humans is of great cost and low efficiency, in the past few decades, the growing demand for objective assessment methods in practical applications has become prominent and urgent. Objective IQA tasks can be divided into three categories—full-reference IQA (FR-IQA), reduced-reference IQA (RR-IQA), and no-reference IQA (NR-IQA)—among which NR-IQA is the most common and challenging method. In the case of NR-IQA, we can only accomplish the IQA task with degraded images, since the pristine images are usually unavailable. Although a great number of IQA algorithms in the last two decades have emerged to achieve a common goal, which is to conform the computational evaluation to human perception, they can only cover limited application requirements we usually meet in practice. Hence, there are still huge potentials and an important gap that needs to be filled in NR-IQA issues.

Early NR-IQA models commonly operate under the hypothesis that images are degraded by particular kind or several specified kinds of distortions [6,7,8,9], which requires a priori knowledge of the image distortion types. Limited by the selection of distortion types, such algorithms depending on the priori cannot achieve further progress. Later, a new NR-IQA class with no demand for prior knowledge of distortion types, labeled as blind image quality assessment (BIQA), appeared. The main idea of BIQA is training a model on the database that consists of distorted images associated with subjective assessment scores. A typical model is the Blind Image Quality Index (BIQI) [10]. With a pre-trained estimation model of distortion types, first, the BIQI method extracts the scene statistics from a given test image. Then, these statistics are used to determine which distortion type(s) the image suffered from, and the final image quality score is computed based on the extracted scene statistics and the pre-judged distortion types. The BIQI model was later extended to the DIIVINE [11] model. The improvement lies in its adoption of a more abundant set of natural scene statistics. Beside of BIQI and DIIVINE, Saad et al. successively proposed two models called BLINDS [12] and BLINDS-II [13]. Both methods can be simplified to learn a probabilistic model from the natural scene statistics-based feature set, and the difference between them is the computational complexity of feature extraction. Moreover, Mittal et al. proposed a model called BRISQUE [14], where they applied locally normalized luminance coefficients to estimate the loss of naturalness of the degraded image and gave a final image quality assessment score on the basis of the loss measurement. Ye et al. [15] proposed an unsupervised feature learning framework for BIQA namely CORNIA, which operates under a coding manner and realizes a combination of feature and regression training. CORNIA was later refined to the semantic obviousness metric (SOM) [16], where object-like regions are mainly detected and processed. In [17], another new BIQA model named DESIQUE was presented, which adopts features in both spatial and frequency domain.

However, all these approaches share the common problem of weak generalization ability [18]. Specifically, these models need to be trained on certain distorted image database(s) to learn a regression model. When applied to other different databases, they show rather weak performance [18]. What is more, since the distortion types are changeable and numerous in the real world and an image can suffer from a single or multiple distortions, it is impossible for a BIQA algorithm to train on a database perfectly containing all such distortion types. In other words, in no way can we acquire complete prior knowledge of image distortion types, which will inevitably result in the poor generalization ability of such algorithms. Therefore, it is of great significance to develop more general and more practical BIQA methods.

The Natural Image Quality Evaluator (NIQE) model [19] possesses better generalization ability. The NIQE model needs to be first trained on a corpus of high-quality images to learn a reference multivariate Gaussian (MVG) model. Then, with a given test image, the NIQE model extracts an NSS-based feature set and fits the feature vectors to an MVG model. Finally, the overall quality of the test image is predicted by measuring the distance between its MVG model and the reference model. However, this method may cause a loss of useful information. Since only one MVG model is used to characterize the test image, some local information of the image is neglected. To tackle this problem, Zhang et al. proposed a new model—IL-NIQE [18]. The IL-NIQE model partitions a test image into patches and extracts an enriched feature sets from each patch. Therefore, a set of MVG models is obtained and the final image quality score is computed by an averaging pooling. Proposed by Bosse et al. [20], a purely data-driven end-to-end deep neural networks for NR-IQA and FR-IQA also takes the advantage of local information and extracts the deep feature with 10 convolutional layers and five pooling layers and performs regression with two fully connected layers. Recently, a new method called MUSIQUE [21] was proposed, which is different from the previous methods that was only applicable to singly distorted images. The MUSIQUE model was designed for both single and multiple distortions applications through a way of estimating three distortion parameters with the NSS-based features and mapping the parameters into an overall image quality score.

However, we observed that these methods obtained unsatisfying results when applied to images with the content of remote sensing scenes (refer to Section 5 for more information), which shows a weak generalization ability to diverse tasks. Therefore, in this work, we seek a method to efficaciously evaluate the quality of images for both real-life natural scenes and RS scenes.

Note that, in this paper, the natural image represents the images of real-life scenes, and the remote sensing image represents that of RS scenes, so as to differentiate the image content as well as to conform to the RGB image format of these two kinds of images.

### 1.2. Our Contributions

**Figure 2:**
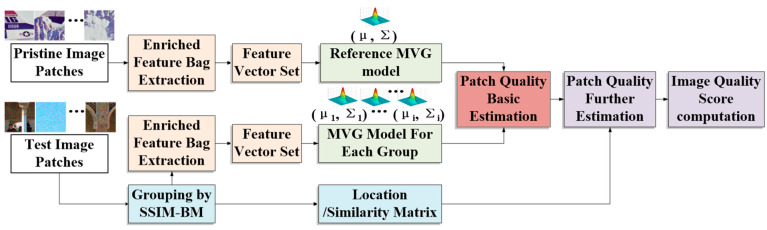
A general framework of the proposed BM-IQE model.

In this paper, we propose a general-purpose BIQA model for real-life scenes as well as remote sensing scenes namely BM-IQE. Inspired by [18] and based on IL-NIQE, we introduced an enriched feature bag (EFB) and a structural similarity block-matching (SSIM-BM) strategy to ensure the proposed method performs well on RS-IQA applications. Meanwhile, the proposed method can achieve competitive performance on natural images as compared to existing state-of-the-art BIQA methods. A general framework of the proposed BM-IQE model is shown in Figure 2.

The contributions of our BM-IQE model are as follows:

1. Datasets for BIQA of the RS scene images are first constructed based on public scene datasets and simulated degraded images.
2. Imaging performance evaluation by means of image quality assessment of the remote sensor is first studied, and a new way of indirectly evaluating the imaging performance of remote sensors is presented.
3. We introduce a block-matching strategy to assist the image patch strategy. This operation can better express the intrinsic features of the image patches (such as affinity, correlation, etc.), as well as to make sure the quality prediction suffering less from image degradations. In this way, the image quality assessment can acquire higher efficacy and accuracy.
4. We adopt four classic Gray-level co-occurrence matrix (GLCM) statistics as texture features to ensure that our method can be more appropriately applied to remote sensing applications comparing with existing IQA models, therefore making sure that the proposed model has an enhanced universality and practicality.

We conducted an extensive series of experiments on various largescale public databases, including RS scene image datasets, singly distorted image, databases and multiply distorted image databases. Note that in many remote sensing applications, such as scene classification and target detection, because images with visible bands of red, green, and blue can fully present the color, texture and contour features of the land cover from the human visual perception aspect, people usually use RGB images for content understanding. Besides, the algorithm proposed in this paper is a general image quality assessment approach, which is oriented to a wide range of natural scene types including both RS scenes and common real-life scenes, so the RGB colors play a very fundamental and significant role as a low-level image feature to recognize objects and understand content. Thus, in this paper, only images with RGB channels are used for experiments, and overall the proposed algorithm was accordingly designed for RBG images of RS scenes and real-life scenes. Experimental results show that the proposed BM-IQE method outperforms other state-of-the-art IQA models on RS scenes and is highly efficacious and robust on real-life scenes.

The rest of this paper is organized as follows. Section 2 introduces the block-matching strategy used in BM-IQE. Section 3 introduces the features we adopt to predict image quality. Section 4 illustrates how the proposed new model is designed. Section 5 presents the experimental results and Section 6 presents the general conclusions of this paper.

## 2. The Proposed Framework

Zhang et al. [18] indicate that partitioning a test image into several patches can make better use of image local features, therefore minimizing the loss of useful information. However, this operation can only enhance the expression of individual patch features but cannot exploit and utilize the latent feature information between the image patches. What is more, the distribution of the degradation effects is uneven on an image, so that the variation caused by degradation in the natural scene statistics extracted from one patch may have a big discrepancy with others. Thus, the statistics calculated from a single patch does not have strong representativeness, and the final pooling result may not well estimate the distortion of the entire image. To mine the correlations among the image patches and make more representative the statistical features of each patch, in this paper, block-matching is introduced as an efficient means to group the similar patches of an image to perform a better feature extraction. By grouping the similar patches, first, the potential local information among different patches can be expressed through group-level feature extraction. Then, the impact on fitting features to an MVG model caused by distortion can be attenuated; the reason is as the data of natural scene statistics increased, so the image NSS feature can realize an enhanced expression and therefore suffer less effects from the distortions.

### 2.1. Grouping

Grouping can be realized in many ways, such as self-organizing maps [22], fuzzy clustering [23], vector quantization [24], etc. However, these methods are computationally demanding and may produce groups with overlaps. Furthermore, in [25], the author indicates that the clustering can cause unequal treatment of different fragments, because the ones that are close to the centroid in the group are more representative than those far from it. Therefore, a more efficient and precise way to realize grouping is needed.

### 2.2. Block-Matching

Block-matching is a strategy often used for image denoising and motion estimation. Matching is a method for finding fragments that are similar to the reference. It can be achieved by estimating the similarity between the reference fragment and each of the candidate fragments. Typical matching models are mean absolute differences (MAD) [26], sum of absolute differences (SAD) [27], sequential similarity detection algorithm (SSDA) [28], sum of absolute transformed difference (SATD) [29], etc. Compared with the grouping methods above, matching methods can achieve grouping in a much more effective and efficient way. Usually, similarity is measured by the distance between two fragments, and the fragments whose distance from the reference is smaller than a specified threshold are considered mutually similar and are subsequently grouped [25]. However, the commonly used distance measurement index such as Euclidean distance and Manhattan distance both have the following inadequacies. First, they are easily affected by the dimension of the data. Second, they cannot effectively reflect the intrinsic correlation of the data [30,31]. Therefore, aiming at avoiding such problems, in this paper, we introduce a new matching model called SSIM-BM, where we take the structural similarity index as the distance metric.

SSIM is a widely accepted FR-IQA metric that comprehensively integrates local luminance, local contrast, and image structure [32]. Then, the similarity is computed by a comprehensive integration of local luminance, local contrast, and structure.

**Figure 3:**
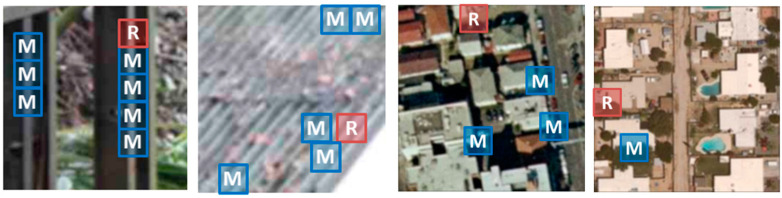
Illustration of grouping blocks from degraded natural images/RS scene images corrupted jointly by Gaussian blur and JPEG compression. Each fragment shows a reference block marked with “R” and a few of the blocks matched to it with “M.”.

Natural images are statistically highly structured, which has been confirmed in both the spatial and frequency domain in previous research. Especially in the case of RS images, they are remarkably characterized by structure features and texture features that convey important information that is useful for human visual perception. Hence, in order to make use of structure-level features, we take SSIM as distance metric for block-matching, where we compare local patterns of pixel intensities that have been normalized for luminance and contrast. The computations of local luminance$\;\mu_{X}$, local contrast $\sigma_{X}$ and local structure$\;\sigma_{XY}$ are given by Equations (1)–(4), respectively.

(1) $$\mu_{X}=\frac{1}{R*C}\sum_{i=1}^{R}\sum_{j=1}^{C}X\left(i,j\right),$$

(2) $$\sigma_{X}^{2}=\frac{1}{R*C-1}\sum_{i=1}^{R}\sum_{j=1}^{C}{\left(X\left(i,j\right)-\mu_{X}\right)}^{2},$$

(3) $$\sigma_{X}=\sqrt{\sigma_{X}^{2}},$$

(4) $$\sigma_{XY}=\frac{1}{R*C-1}\sum_{i=1}^{R}\sum_{j=1}^{C}\left(X\left(i,j\right)-\mu_{X}\right)\left(Y\left(i,j\right)-\mu_{Y}\right),$$

where *i* and *j* are spatial coordinates and R and C represent the size of input data. In this way, we can more efficaciously exploit the potential similarity so that estimate the true signals among the image fragments. An illustration of block-matching and grouping is presented in Figure 3.

Note that to determine the threshold for SSIM-BM, we observe the grouping results after each pass of BM test and make corresponding adjustment. To this purpose, the grouping accuracy (higher value leads to small group scale) and the group scale need to be balanced, thus the tuning criterion is set as the threshold value leading to no false positive result and no less than two patches (include the reference patch) in each group is chosen.

By grouping similar image patches together, we can obtain an increasing amount of feature data through group-level feature extraction. Hence, the feature vectors can be more soundly represented and the true signals among different patches can get expressed. The products of SSIM-BM, beside those of the matrix made up of similar patch groups, include a similarity matrix and a location matrix. The former records the similarity computed during each matching operation, and the latter records the location of each matched patch in the given test image. Thus, both of the matrices have the same size with the group array, and their elements correspond one-to-one. All these products will be later used for a pooling strategy.

## 3. NSS Feature Extraction

In previous works, NSS-based features have shown great image quality prediction abilities, which makes the features in natural image BIQA tasks widely used. The NSS-based features can be extracted from spatial domain [11,12], DCT domain [13,14], Wavelet domain [33], etc. Each different NSS feature delivers useful information from an aspect of images. For RS images, they are characterized by the spectrum features, edge features and texture features. However, the existing general-purpose BIQA models mainly focus on the grayscale features and structure features but rarely discuss about the image texture feature [18,21]. Therefore, in order to improve the evaluation performance and enlarge the application scope of the general-purpose BIQA models, in BM-IQE, we introduce texture features to better conform to the remote sensing scopes. Although some of the features we adopt have been introduced in previous works, we collectively propose a new feature bag consisting of texture features and several existing features. Experiments show that the newly introduced feature bag possesses superior image quality prediction performance.

### 3.1. Optics Features Extraction

#### 3.1.1. Statistical Features of MSCN Coefficients

Human visual perception is sharp to the areas in an image of high contrast. Changes in image contrast can have significant impacts on the image quality. Hence, as the image contrast is closely related to both of the subjective and objective image quality evaluation, we adopt the contrast feature for our BIQA tasks. The mean subtracted contrast normalized (MSCN) coefficient is a kind of commonly used contrast feature. Ruderman et al. [34] pointed out that the locally normalized luminance map of a natural grayscale photographic image *I* conforms to a Gaussian distribution. The products of the normalized process are called MSCN coefficients, and the normalized process is given by:

(5) $$\hat{I}\left(i,j\right)=\frac{I\left(i,j\right)-\mu \left(i,j\right)}{\sigma \left(i,j\right)+1},$$

(6) $$\mu \left(i,j\right)=\sum_{k=-K}^{K}\sum_{l=-L}^{L}\omega_{k,l}I_{k,l}\left(i,j\right),$$

(7) $$\sigma \left(i,j\right)=\sqrt{\sum_{k=-K}^{K}\sum_{l=-L}^{L}\omega_{k,l}{\left(I_{k,l}\left(i,j\right)-\mu \left(i,j\right)\right)}^{2}},$$

where *i* and *j* are spatial coordinates. $\omega =\{\omega_{k,l}|k=-K,\ldots ,K,l=-L,\ldots ,L\}$ defines a unit-volume Gaussian window. *μ* is the local mean field, and *σ* is the corresponding local variance field. *μ* and *σ* denote the image local mean and image local contrast, respectively. An important attribute of MSCN coefficients is that its local correlation over the image content is not strong. Therefore, we can ensure the employed features take effects over different image scenes.

The MSCN coefficients map is proved to conform to a unit Normal distribution. According to (5)–(7), the luminance map I of a test image is decorrelated through local mean subtraction and divisive normalization process to yield the MSCN coefficients map. Then, a zero-mean generalized Gaussian distribution (GGD) is adopted to model the histogram of the MSCN coefficients map $\hat{I}\left(i,j\right)$, and its density function is given by

(8) $$g\left(x;\alpha ,\beta \right)=\frac{\alpha }{2\beta \Gamma \left(\frac{1}{\alpha }\right)}exp\left(-{\left(\frac{\left|x\right|}{\beta }\right)}^{\alpha }\right),$$

where

(9) $$\beta =\sigma \sqrt{\frac{\Gamma \left(\frac{1}{\alpha }\right)}{\Gamma \left(\frac{3}{\alpha }\right)}},$$

and Γ(⋅) is the gamma function

(10) $$\Gamma \left(x\right)=\int_{0}^{\infty }t^{x-1}e^{-t}dt,x>0,$$

The parameter α takes control of the shape of the GGD, and β controls the corresponding variance. The two parameters are employed as NSS features for image quality prediction.

In addition to the MSCN coefficients histogram, some of its derived NSS features were also introduced in previous works. In [14], the statistical relationships between neighboring pixels were modeled. The author suggested that while MSCN coefficients are definitely more homogenous for pristine images, the signs of adjacent coefficients also exhibit a regular structure, which gets disturbed in the presence of distortion. Pairwise neighboring MSCN coefficients are computed along four orientations: horizontal (H), vertical (V), main-diagonal (MD) and secondary-diagonal (SD); they are denoted by $\hat{I}\left(i,\;j\right)\hat{I}\left(i,\;j+1\right)$, $\hat{I}\left(i,\;j\right)\hat{I}\left(i+1,\;j\right)$, $\hat{I}\left(i,\;j\right)\hat{I}\left(i+1,\;j+1\right)$, and $\hat{I}\left(i,\;j\right)\hat{I}\left(i+1,\;j-1\right)$, respectively, where *i* and *j* are the spatial coordinates.

The paired products of neighboring MSCN coefficients are observed to follow a zero-mode asymmetric generalized Gaussian distribution (AGGD),

(11) $$f\left(x;\gamma ,\beta_{l},\beta_{r}\right)=\{\begin{array}{c} \frac{\gamma }{\left(\beta_{l}+\beta_{r}\right)\Gamma \left(\frac{1}{\gamma }\right)}exp\left(-{\left(\frac{-x}{\beta_{l}}\right)}^{\gamma }\right),\;\forall x\leq 0.\\ \frac{\gamma }{\left(\beta_{l}+\beta_{r}\right)\Gamma \left(\frac{1}{\gamma }\right)}exp\left(-{\left(\frac{x}{\beta_{r}}\right)}^{\gamma }\right),\;\forall x\geq 0. \end{array},$$

The mean of the AGGD is

(12) $$\eta =\left(\beta_{r}-\beta_{l}\right)\frac{\Gamma \left(\frac{2}{\gamma }\right)}{\Gamma \left(\frac{1}{\gamma }\right)},$$

Then, the parameters $\left(\gamma ,\;\beta_{l},\;\beta_{r},\;\eta \right)$ are taken as derived NSS features at four orientations for our BIQA tasks. Particularly, all MSCN statistical features are extracted at two scales (the original scale and a low-pass down-sampled scale) to capture multi-scale information.

#### 3.1.2. Statistical Features of Colors

In order to exploit further information that human perception is closely related to in color images, we resort to a classical NSS model. Ruderman et al. [35] pointed out that the distributions of natural image statistics conform well to a Gaussian probability model in a logarithmic-scale opponent color space. The color values (R, G, B) for each pixel in an image are determined from three human cone quantal catches through a direct linear correspondence (refer to [36] for detail), and the specifical coordinate transformation is as follows. Each of the three channels R(i, j), G(i, j), and B(i, j) is converted to a logarithmic signal, where the mean is subtracted away:

(13) $$R\left(i,j\right)=\log R\left(i,j\right)-\mu_{R},$$

(14) $$G\left(i,j\right)=\log G\left(i,j\right)-\mu_{G},$$

(15) $$B\left(i,j\right)=\log B\left(i,j\right)-\mu_{B},$$

where$\;\mu_{R}$, $\mu_{G}$ and $\mu_{B}$ are the corresponding mean values to the logR(i, j), logG(i, j) and logB(i, j) over the image. Then, an orthogonal decorrelation process is added to the three logarithmic signals robustly producing three principal axes, which are given by:

(16) $$\;l_{1}\left(i,\;j\right)=\left(R+G+B\right)/\sqrt{3},$$

(17) $$l_{2}\left(i,\;j\right)=\left(R+G-2B\right)/\sqrt{6},$$

(18) $$\;l_{3}\left(i,\;j\right)=\left(R-G\right)\sqrt{2},$$

The coefficients$\;l_{1}$, $l_{2}$, and $l_{3}\;$are observed to follow a Gaussian and symmetrical distribution. The empirical density functions of$\;l_{1}$, $l_{2}$, and$\;l_{3}$ are given by a Gaussian model,

(19) $$f\left(x;\mu ,\sigma^{2}\right)=\frac{1}{\sqrt{2\pi }\sigma }exp\left(\frac{-{\left(x-\mu \right)}^{2}}{2\sigma^{2}}\right),$$

By estimating the parameters μ and$\;\sigma^{2}$, we can obtain two additional NSS features and the features are extracted for each of the three channels.

### 3.2. Structure Features Extraction

Generally speaking, the optics features we introduced in Section 3.1 can provide amounts of useful information. However, the calculations of these features are mainly made up of individual pixels, which restricts the features to the “unstructured” aspects of the image—equivalent to an investigation of individual rays of light [37]. As T. Pouli pointed out, the first and most obvious way to look at image structure is to examine the relationship between pairs of pixels. Gradient is such a functional statistic. As is given by (20)–(21), the gradients at pixel (i, j) are calculated by convolving the luminance map I with the Gaussian derivative filter along horizontal and vertical orientations, respectively:

(20) $$D_{i}=I\left(i,\;j\right)⊗\frac{\partial G\left(i,j;\sigma \right)}{\partial i},$$

(21) $$D_{j}=I\left(i,\;j\right)⊗\frac{\partial G\left(i,j;\sigma \right)}{\partial j},$$

where G(i, j) is a two-dimensional Gaussian distribution expressed as

(22) $$G\left(i,j;\sigma \right)=\frac{1}{2\pi \sigma^{2}}e^{\frac{-\left(i^{2}+j^{2}\right)}{2\sigma^{2}}},$$

**Figure 4:**
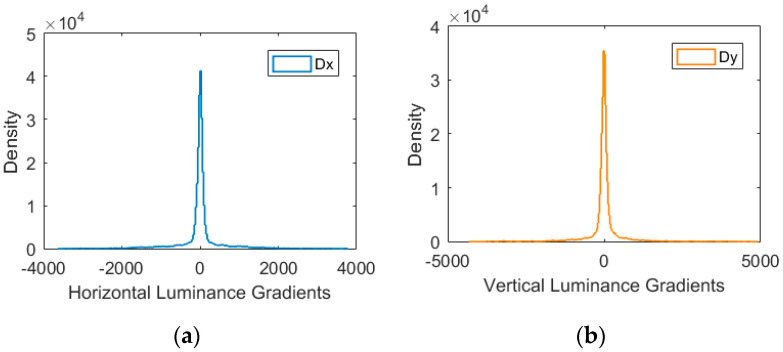
An example of gradient distributions for a natural image. (**a**) Dx represents the horizontal gradients (first derivative of the intensity function); (**b**) Dy represents the vertical gradients (first derivative of the intensity function).

$D_{i}$ is the horizontal gradient and $D_{j}$ is the vertical gradient. It has been found that natural image gradients are well modeled by a GGD distribution [18]. An example of the gradients distributions for an image is given by Figure 4, where $D_{x}$ and $D_{y}$ denotes the horizontal and vertical gradients. Therefore, by fitting the histograms of the gradient components $D_{i}$ and $D_{j}$ to the GGD model, we can estimate the parameters (α,β) and adopt them as quality aware features.

Beside of the gradient components, in both cases, it is common to calculate the mean gradient magnitude at a given location from the horizontal and vertical components [37]:

(23) $$D\left(i,j\right)=\left|\begin{array}{c} D_{i}\\ D_{j} \end{array}\right|=\sqrt{D_{i}{}^{2}+D_{j}{}^{2}},$$

The gradient magnitude of natural images conforms well to a Weibull distribution:

(24) $$p\left(x;a,b\right)=\{\begin{array}{ll} \frac{a}{b^{a}}x^{a-1}exp\left(-{\left(\frac{x}{b}\right)}^{a}\right), & x\geq 0.\\ 0, & x\leq 0. \end{array},$$

where the parameters *a* and *b* control the shape and scale of the Weibull distribution, respectively. Recent studies in neuroscience suggested that the responses of visual neurons are strongly correlate with Weibull statistics during the image processing [38]. Hence, with an optimal fitting of the image gradient magnitude histogram to the Weibull distribution, we obtain the two parameters *a* and *b* as NSS features.

In order to further investigate the expression of distortions in image color space, we map the RGB images into a perceptually relevant opponent color space, and the weights in the conversion are perceptually optimized on human visual statistics [39]:

(25) $$\left[\begin{array}{c} O_{1}\\ O_{2}\\ O_{3} \end{array}\right]=\left[\begin{array}{ccc} 0.06 & 0.63 & 0.27\\ 0.30 & 0.04 & -0.35\\ 0.34 & -0.6 & 0.17 \end{array}\right]\left[\begin{array}{c} R\\ G\\ B \end{array}\right],$$

Based on this, the gradient components and derived magnitude features are also computed on each channel of${\;O}_{1}$, $O_{2}$, and ${\;O}_{3}$ as NSS features.

### 3.3. Texture Features Extraction

#### 3.3.1. Statistics of GLCM

Texture analysis has been widely used in remote sensing image interpretation and processing. Textures in an image are generally understood as a repetitive arrangement of some basic patterns, which can to some extent reflect the structural characteristics of objects. The main idea of texture analysis is to specify the spatial distribution patterns of grayscale images, and it can be effectively realized by means of the GLCM.

GLCM is commonly used for remote sensing image analysis. It utilizes the spatial distribution of gray levels to describe the image texture. Since the gray level distribution are significantly influenced by the presence of distortions, we take GLCM as a powerful analysis tool in our BIQA tasks.

**Figure 5:**
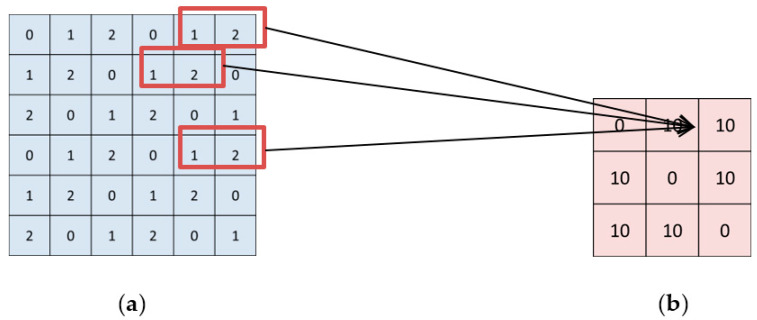
A possible example of luminance map and associated Gray-level co-occurrence matrix (GLCM) map of an image. (**a**) is the luminance map *I*; (**b**) is the GLCM map.

Figure 5 is an illustration of the GLCM calculation. Given the image luminance map *I*, we can obtain the corresponding GLCM map. Specifically, from Figure 5b we can see the GLCM value at the coordinate (1,1) is 0, which means that no adjacent pixel pair with gray values of (1, 1) can be found in the luminance map (Figure 5a). Likewise, the GLCM value at the coordinate (1, 2) is 10, which means that 10 pairs of adjacent pixels with gray values of (1, 2) can be found in the image. In general, GLCM map is essentially all possible combinations of adjacent gray values, where the adjacent can be understood in different directions. When the pixel pair is given by f(i, j) and f(i+1, j), we regard the pixels as adjacent in the 0° orientation. When the pixel pair is given by f(i, j) and f(i, j+1), we regard the pixels as adjacent in the 90° orientation. When the pixel pair is given by f(i, j) and f(i+1, j+1), we regard the pixels as adjacent in the 45° orientation. When the pixel pair is given by f(i, j) and f(i−1, j+1), we regard the pixels as adjacent in the 135° orientation. Hence, the GLCM map is computed at four orientations with three dimensions for color images.

After we obtain the GLCM map, derived statistics can be further computed according to the map. In this work, we introduce four classic statistics of the GLCM map—contrast, energy, entropy, and correlation. The contrast statistic is closely related to the definition and the texture depth of the image. The deeper the texture groove, the larger the contrast values, and the image is correspondingly clearer. The energy statistic reflects the uniformity of gray levels distribution which essentially represents the texture fineness. A uniform GLCM map represents a fine texture pattern producing a smaller energy value, and an uneven GLCM map represents a coarse texture pattern yielding a larger energy value. The entropy statistic measures the image information randomness. When the values in the GLCM map are all equal, the image pixels show the greatest randomness and the entropy reaches the maximum. Furthermore, the entropy shows the complexity of image gray levels distribution. The larger entropy value suggests a more complex image structure. Correlation is often used to measure the similarity of gray levels in row or in column directions, and a larger correlation indicates a greater image gray levels similarity.

The calculations of the four GLCM statistics are given by

(26) $$Con=\sum_{i}\sum_{j}{\left(i-j\right)}^{2}P\left(i,j\right),$$

(27) $$Eng=\sum_{i}\sum_{j}P{\left(i,j\right)}^{2},$$

(28) $$Ent=-\sum_{i}\sum_{j}p\left(i,j\right)\log p\left(i,j\right),$$

(29) $$Cor=\frac{\sum_{i}\sum_{j}\left(\left(ij\right)p\left(i,j\right)\right)-\mu_{i}\mu_{j}}{\sigma_{i}\sigma_{j}},$$

where i, j are the spatial coordinates.

To testify that the four statistics introduced above are effective and useful NSS features for the image quality prediction, we conducted a set of demonstrative experiments and the scheme is as follows.

1. Compute the GLCM map and four derived statistics on a high-quality test image.
2. Use the 5×5 Gaussian kernel to blur the pristine image. The deviation of Gaussian filters is set to four levels—0, 0.5, 1, and 5. The GLCM map and four derived statistics are computed on each blurred image.
3. Add white noise to the pristine image with zero mean and various variances. The variance of white noise is set to four levels—0, 0.001, 0.005, and 0.01. The GLCM map and four derived statistics are also computed on each degraded image.

**Figure 6:**
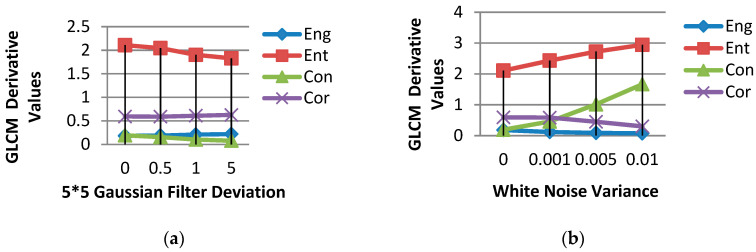
The GLCM-derived statistics of distorted images. (**a**) presents the results of images distorted by Gaussian blur distortion; (**b**) presents the results of images distorted by white noise distortion. As shown on the *x*-axis, four distortion levels were set for each distortion type.

Figure 6 presents the experiment results. It shows that the four GLCM derivatives can keep great consistency and linearity under varying degradations, which suggests that the four statistics are useful and effective for image quality prediction tasks. Hence, we adopt them as quality-aware features.

#### 3.3.2. Statistics of Log-Gabor Filter Response

Previous research proved that visual cortex neurons respond selectively to stimulus at disparate orientations and frequencies [18,40]. Hence, the multi-scale and multi-orientation filter responses to the image are also significant information for quality assessment tasks.

Fourier transform is known as a powerful tool for signal processing. However, a noticeable disadvantage of the Fourier transform is that the same frequency image elements at different spatial locations are often mixed together, which makes it impossible to achieve a local multi-scale and multi-orientation analysis. Different from Fourier transform, the Gabor filter can extract specified frequency and orientation information from local spatial fields, therefore making itself an effective texture analysis tool.

In order to better take use of the characteristics of Gabor filter, in this work, we deploy perceptually relevant log-Gabor filters [40] with multiple orientations and scales to accomplish filtering. A 2D log-Gabor filter is given by

(30) $$G_{2D}\left(\omega ,\theta \right)=e^{-\frac{{\left(\log\left(\frac{\omega }{\omega_{0}}\right)\right)}^{2}}{2\sigma_{r}{}^{2}}}\times e^{-\frac{{\left(\theta -\theta_{j}\right)}^{2}}{2\sigma_{\theta }{}^{2}}},$$

where $\omega_{0}$ is the center frequency. $\theta_{j}=j\pi /J$ is the orientation angle, j={0, 1, …, J−1} is the orientation factor, and J is the number of orientations. $\sigma_{r}\;$and $\sigma_{\theta }$ control the radial bandwidth and the angular bandwidth of the filter, respectively. With log-Gabor filters possessing *N* different center frequencies and *J* different orientations applied to an image f(x), we can acquire a set of 2*NJ* responses$\;\left\{R_{n,j}\left(x\right),\;I_{n,j}\left(x\right),n=0,\ldots ,N-1,\;j=0,\ldots ,\;J-1\right\}$, where $R_{n,j}\left(x\right)$ and $I_{n,j}\left(x\right)$ are the real and imaginary components of the log-Gabor filters responses, respectively. As the filter responses maps are observed to follow a Gaussian distribution, we use the GGD to model the distributions of $\left\{R_{n,j}\left(x\right)\right\}\;$and $\left\{I_{n,j}\left(x\right)\right\}$,

(31) $$g\left(x;\alpha ,\beta \right)=\frac{\alpha }{2\beta \Gamma \left(\frac{1}{\alpha }\right)}exp\left(-{\left(\frac{\left|x\right|}{\beta }\right)}^{\alpha }\right),$$

The best-fit parameters (α, β) are taken as quality prediction features.

Besides, we use the GGD to model the distributions of the smoothed directional gradient components of $\left\{R_{n,j}\left(x\right)\right\}\;$and $\left\{I_{n,j}\left(x\right)\right\}$, and the best-fit parameters are extracted as NSS features. Furthermore, we use the Weibull distribution (Equation (24)) to model the smoothed directional gradient magnitudes of $\left\{R_{n,j}\left(x\right)\right\}\;$and $\left\{I_{n,j}\left(x\right)\right\}$, and take the best-fit parameters (a,b) as NSS features.

## 4. Algorithm

The BM-IQE model was constructed in two stages—the training stage and the testing stage. In the training stage, we learn a reference multivariate Gaussian (MVG) model of the NSS-based features from a collection of pristine images. Then, in the testing stage, similar patches of a given test image are grouped through block-matching and a feature vector is extracted from each group. For each feature vector, an MVG model is fitted to and the basic quality estimation of each patch (centroid of the group) is given by measuring the distance between group MVG model and the reference MVG model. Further quality estimation of each patch is obtained by weighting averaging of all its similar patches’ basic estimations. Finally, the overall image quality of the test image is computed by an average pooling of the patch quality estimations.

### 4.1. Reference MVG Model Learning

Inspired by the IL-NIQE, we learn a reference MVG model to characterize the statistical NSS features of natural pristine images. We collected online a total of 100 high quality natural pristine images, which cover people, animals, architectures, plants, etc. Only images generally recognized as high-quality images are eventually selected. Note that experiments were conducted by adopting different size of image set for training, which demonstrates that 100 images are enough to produce a sound reference model. Besides, there is no overlap between the selected pristine images and the IQA databases that will be later used in algorithm performance evaluation, which ensures the effectiveness of our BIQA method.

After the natural pristine image corpus created, we started with learning the reference MVG model. First, we partitioned the pristine images into patches of size p×p, and the high-quality patches are selected out. Then, the EFB we introduced in Section 3 were extracted from each patch yielding a feature vector $\;x_{i}$. As some features can be correlated with each other, such as the gradient components and magnitude features, we applied PCA to the feature vector to reduce the computational cost. The PCA can be described as

(32) $$x_{i}^{\prime }=\Phi^{T}x_{i},\;x_{i}^{\prime }\in R^{m\times 1},i=1,\ldots ,n,$$

where $x_{i}$ are the elements of $X=\left[x_{1},x_{2},\ldots ,x_{n}\right]\in R^{d\times n}$ the matrix of feature vectors. $\Phi \in R^{d\times m}$ is the learned projection matrix, of which the dimension m is determined by solving the following error function:

(33) $$1-\frac{\sum_{1}^{m}S_{i}}{\sum_{1}^{d}S_{i}}\leq 0.01,$$

where S denotes the eigenvalue matrix, d denotes the original dimension, m denotes the wanted dimension. As a result of PCA, we obtained $\left\{x_{i}^{\prime },\;i=1,\ldots ,n\right\}$ as samples for the MVG distribution. The final reference MVG model is given by

(34) $$f\left(X^{\prime }\right)=\frac{1}{{\left(2\pi \right)}^{\frac{m}{2}}{\left|\Sigma \right|}^{\frac{1}{2}}}exp\left(-\frac{1}{2}{\left(X^{\prime }-\mu \right)}^{T}\Sigma^{-1}\left(X^{\prime }-\mu \right)\right),$$

where $X^{\prime }$ is the vector variable, and (μ, Σ) are the mean vector and the covariance matrix of $X^{\prime }$, respectively. The reference MVG model can be fully described by the parameters (μ, Σ).

### 4.2. Distorted Image Quality Prediction

Like the pristine images in the training stage, the given test image is partitioned into patches of size p×p and these patches are numbered in sequence. Then, similar patches are grouped together through SSIM-BM. Specifically, each patch is used as a reference patch and the SSIM metrics are computed between the reference patch and all the other patches. The patches with greater metric values (smaller distance from the reference patch) than a given threshold are considered mutually similar and subsequently grouped together. For each group, the reference patch can be regarded as the “centroid” of this group; the sequence numbers and metric values of other patches are memorized in a location matrix $M_{LOC}$ and similarity matrix $M_{SIM}$ respectively for later use. Then, the NSS feature vector $y_{i}$ is extracted from each group, and the feature vector $y_{i}^{\prime }$ after PCA can be easily obtained using the pre-learned projection matrix Φ:

(35) $$y_{i}^{\prime }=\Phi^{T}y_{i},y_{i}^{\prime }\in R^{m\times 1},i=1,\ldots ,k,$$

Extensional experiments have been made to verify the validity of the use of Φ for distorted images. After obtaining Φ in training stage, arbitrary images are given to apply PCA to. The error function (33) was used to measure the information loss of PCA, and thus, was computed between the feature vectors before and after PCA operation. The test results turn out to be all below 0.05. Therefore, the projection matrix is subjectively assumed to be scalable.

**Figure 7:**
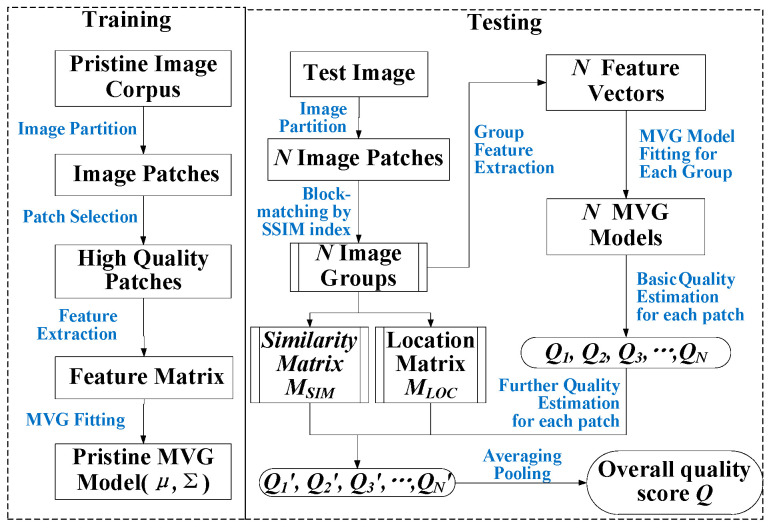
Processing flow of the BM-IQE model. The training stage is required to run before the testing stage. The output model (*μ*, Σ) of the training stage is used for the calculation of *Q_i_* in the testing stage.

After obtaining the feature vector set $\left\{y_{i}^{\prime },\;i=1,\;\ldots ,\;n\right\}$, an MVG model denoted by $\left(y_{i}^{\prime },\Sigma^{\prime }\right)$ is fitted to each group, where $y_{i}^{\prime }$ means $\mu_{i}$ and the empirical covariance $\Sigma^{\prime }$ of {$y_{i}^{\prime }$} means ${\;\Sigma }_{i}$. By comparing the group MVG model against the reference MVG model, we can obtain the basic quality estimation $Q_{i}$ of the *i*-th group centroid patch. The basic quality score is given by

(36) $$Q_{i}=\sqrt{{\left(\mu -y_{i}^{\prime }\right)}^{T}{\left(\frac{\Sigma +\Sigma^{\prime }}{2}\right)}^{-1}\left(\mu -y_{i}^{\prime }\right)},$$

where (μ, Σ) describes the reference MVG model, $\left(y_{i}^{\prime },\;\;\Sigma^{\prime }\right)$ describes the MVG model of patch *i* (centroid of group *i*). Then, by utilizing the location matrix and similarity matrix we obtained after SSIM-BM, the further quality estimation $Q_{i}^{\prime }$ of each patch *i* is given by a weighted average of all the similar patches’ basic quality estimations, where the similarity is taken as the weight for averaging. Finally, the overall quality score Q of the test image can be computed by an averaging pooling of all the patch quality estimations. The process of computing the overall image quality score is illustrated in Figure 7, and the BM-IQE Algorithm 1 is summarized below.
**Algorithm 1** BM-IQE**Training****Input:** Training image set; Patch size p; Log-Gabor filters parameters.Partition images into patches of size p**for** all image patches   Extract feature vectors from image patches using Equations (5)–(31)**end for**Apply PCA to the feature vectors using Equation (32)Conform the results of PCA to a MVG model using Equation (33)**Output:** The reference MVG model given by (μ, Σ)**Testing****Input:** Testing image I; reference MVG model; Patch size p; Log-Gabor filters parameters; block-matching threshold t.Partition I into patches of size p**for**N image patches**for** the rest N−1 patchesrun global SSIM-BM using Equations (1)–(4)**while** similarity ≥t Update the patch group, the similarity matrix $M_{LOC}$ and the location matrix $M_{SIM}$    **end while**   **end for****end for**Extract feature matrixes from N patch groups using Equations (5)–(31)Apply PCA to the N feature matrixes using Equation (32)Conform the results of PCA to N MVG models respectively using Equation (33)Calculate $Q_{i}$ according to the reference MVG model using Equation (36)Calculate $Q_{i}^{\prime }$: $Q_{i}^{\prime }=\frac{\sum_{N}Q_{i}M_{\mathrm{SIM}}{}_{i}}{N}$Average $Q_{i}^{\prime }$ to produce the overall quality score Q**Output:**Q

## 5. Results and Discussion

In this section, we present the experimental results and analyze BM-IQE’s performance on blind image quality assessment by employing various natural image databases and remote sensing scene datasets as well as comparing it with existing FR/NR-IQA algorithms.

### 5.1. Training Details

Inspired by [18], we tuned the parameters on a subset of the TID2013 database. The subset consists of 10 reference images and associated 1200 distorted images. The tuning criterion is that the parameter value leading to a higher Spearman rank-order correlation coefficient (SRCC) is chosen. In our final completion, we set the patch size p to 84 and the PCA transformed features dimension *m* to 430. The parameters of the log-Gabor filters are set as follows: *N* = 3, *J* = 4, $\sigma_{r}\;$= 0.60, $\sigma_{\theta \;}$= 0.71, $\omega_{0}^{1}$ = 0.417, $\omega_{0}^{2}$ = 0.318, and $\omega_{0}^{3}$ = 0.243, where $\omega_{0}^{1}$, $\omega_{0}^{2}$ and $\omega_{0}^{3}$ represent the three center frequencies of the log-Gabor filters at three scales. Particularly, for the block-matching threshold *t*, we tuned this parameter on a dataset consists of 50 distorted images cover different image content. The testing results were manually inspected to ensure that the entire testing dataset complies with the tuning rule presented in Section 2.2. In the final completion, the threshold *t* was set to 0.69.

### 5.2. Testing Details

To evaluate the prediction ability of BM-IQE model, five general-purpose IQA databases were used—(1) the LIVE database [41], (2) the TID2013 database [42], (3) the CSIQ database [43], (4) the IVC database [44], and (5) the LIVE MD database [45]. Besides, the UC Merced Land Use Dataset [46] and the Aerial Image Dataset (AID) [47] (RS image datasets) were particularly adopted. Among the IQA databases, the LIVE MD and TID2013 databases include multiply distorted images and the remaining three are singly distorted image databases. The information of these databases is summarized in Table 1.

We compare the BM-IQE with eight state-of-the-art FR/NR-IQA models, including (1) SSIM [35], (2) FSIM [48], (3) VIF [49], (4) BRISQUE [14], (5) DIIVINE [11], (6) NIQE [19], (7) IL-NIQE [18], (8) MUSIQUE [21], and (9) WaDIQaM-NR [20]. In the IQA research area, the goodness of any algorithm is gauged by measuring the correlation of algorithmic scores with subjective (scored by human) mean opinion scores (DMOS/MOS) on a large dataset covering different distortion. Thus, three typical metrics were adopted to evaluate the prediction performance of the competing algorithms—(1) the Spearman Rank-Order Correlation Coefficient (SROCC), (2) the Pearson linear correlation coefficient (PLCC), and (3) the Root Mean Square Error (RMSE) were used to measure the rank-order correlation, the linear correlation and the degree of dispersion between two the groups of scores, respectively. The SROCC metric is computed between the objective scores predicted by BIQA algorithms and the subjective mean opinion scores (MOS) provided by images database and is generally used to evaluate the prediction monotonicity and accuracy. The PLCC and RMSE metrics are computed between the subjective and objective scores following a nonlinear regression:

(37) $$f\left(x\right)=\beta_{1}\left(\frac{1}{2}-\frac{1}{1+exp\left(\beta_{2}\left(x-\beta_{3}\right)\right)}\right)+\beta_{4}x+\beta_{5}\;,$$

where $\beta_{i}\;,\;i=1,\;2,\ldots ,\;5$ are the parameters that need to be fitted.

In short, the higher the SROCC and PLCC values and the lower RMSE values, the better the ability of prediction behaves.

### 5.3. Evaluation of Features

In our algorithm, six types of features were employed, including (1) MSCN features, (2) adjacent MSCN features, (3) color features, (4) gradient features, (5) log-Gabor filter response features, and (6) GLCM features. In order to demonstrate the effectiveness and efficiency of each selected feature, we evaluated the performance of each feature on IQA databases. The pre-trained projection matrix remained fixed for all experiments and the SROCC metric was used to evaluate the feature prediction ability. Experimental results are shown in Table 2. 

What is more, since the block-matching is introduced to extract group-level features, a comparative experiment was presented to verify that the expression discrepancy of individual patch features is effectively improved. The SROCC and PLCC metrics were used to measure the algorithm performance. The overall results are reported in Table 3, and the best-performing results are in bold.

From Table 2, we can see that the front five types of features both show poor BIQA performance, nevertheless, the four GLCM features show superior performance on all databases, which suggests that the texture conveys more important information for visual perception evaluation. Besides, among the front five features, the log-Gabor filters response feature performs comparatively better and the color feature performs the worst. It indicates that the selected multi-scale and multi-orientation filters responses feature can effectively characterize image quality, while the color space we adopted is not suitable for the natural image statistical description. More sufficient representative feature models are needed in future works.

From Table 3, we can intuitively summarize that on all employed databases, algorithm with block-matching strategy performs much better than that without block-matching. The only deficiency of our model goes to the PLCC value on the TID2013 database; however, the SROCC metric is worth more for the performance measurement than the PLCC metric, and the SROCC result of our model is numerically slightly better. Therefore, we can conclude that the proposed method with a block-matching strategy is of high effectiveness and great values.

### 5.4. Performance on Remote Sensing Databases

In this section, we analyze the quality prediction abilities of BM-IQE and other FR/NR-IQA models on remote sensing image datasets. Two benchmark datasets were used in experiments. The first one is the UC Merced Land Use Dataset with 21 classes land use images meant for research purposes. There are 100 images for each of the following classes: agricultural, airplane, golf course, beach, buildings, chaparral, dense residential, forest, freeway, harbor, baseball diamond, intersection, medium residential, mobile home park, overpass, parking lot, river, runway, sparse residential, storage tanks, and tennis court. Each image measures 256 × 256 pixels. Similarly, the second one is the Aerial Image Dataset (AID), which has 30 different scene classes and about 200 to 400 samples of size 600 × 600 in each class.

**Figure 8:**
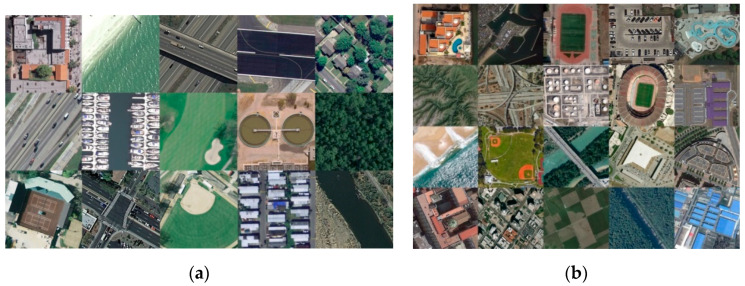
RS images selected from (**a**) the UC Merced Land Use Dataset and (**b**) the Aerial Image Dataset are used for algorithm performance testing.

In this work, we selected 15 pristine RS images of different classes from the UC Merced Land Use Dataset and 20 from the Aerial Image Dataset. The thumbnails of all these images are shown in Figure 8. Note that in order to truly and objectively evaluate the imaging performance of an imaging system, only the degradation types caused by imaging system should be considered regardless of such environmental disturbance as cloud cover or atmospheric turbulence. Particularly, for the band to band mismatch distortion, though caused by the imaging system, it usually existed on remote sensors of early years. However, with the technology of spectral splitting, the geometric deviation between the bands of the multispectral image is negligible. Thus, aiming at the application for current remote sensors, we think there is not much necessity to consider the band to band mismatch distortion. For another distortion type as the dropping line, it is also caused by the imaging system. This kind of image distortion has a noticeable manifestation, and the visual effect of the image will significantly decline under this situation, which can straightforwardly reflect the trouble of remote sensor imaging equipment and the decline of imaging performance. Hence, there is no necessity to quantify the dropping line distortion through the image quality assessment process. Based on above consideration, we select the white noise, Gaussian blur and JPEG compression distortions as subjects to conduct the experiments on RS images.

**Figure 9:**
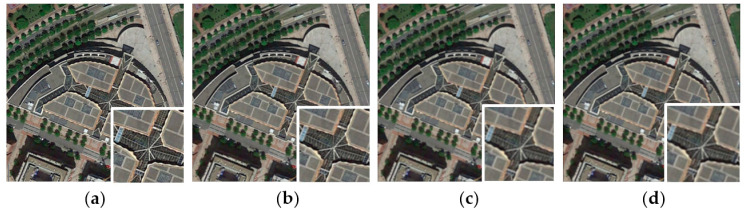
Samples of blur images from the built remote sensing image database. The degradations gradually increased from (**a**–**d**), and the corresponding quality scores obtained with BM-IQE are given as 15.5828, 18.2615, 25.0430, 28.0898, respectively.

We added white noise, Gaussian blur and JPEG compression distortions to each pristine image respectively to set up a degraded image dataset. Each type of distortion was set to five different levels, and the subjective score was determined by the averaging of 10 people’s grading results. Note that the 10 people were all trained on the standard IQA databases to ensure a consistent guideline on their judgments. Six NR-IQA algorithms were tested on the RS image dataset: BRISQUE, DIIVINE, NIQE, IL-NIQE, WaDIQaM-NR, and BM-IQE. SROCC and PLCC were used to measure the quality prediction performance of each IQA model. An example regarding distorted images and corresponding objective scores is given in Figure 9. The overall testing results are reported in Table 4 and Table 5.

From Table 4 and Table 5, we can see that on the RS scene image datasets, BM-IQE shows much better BIQA performance than the other algorithms. For all types of distortions, compared with other models, BM-IQE obtains the best evaluation results and the metric values manifest a great progress on RS-IQA problem. Nevertheless, none of the five models can obtain an actually satisfying result. A possible reason is the texture did not get soundly expressed by existing features, though the GLCM features have effectively improved the IQA performance, they are far from meeting our demands. Hence, the quality assessment of RS scene image deserves further exploration.

### 5.5. Performance on Individual Databases

In this section, we evaluate the quality prediction performance of BM-IQE and other algorithms on individual distortion databases. Three FR-IQA models and five NR-IQA models were used to compare with BM-IQE—SSIM, FSIM, VIF, BRISQUE, DIIVINE, NIQE, IL-NIQE, and WaDIQaM-NR.

First, all algorithms need to train on a subset of the TID2013 database, and this subset will not be used in later testing stage. Then, the algorithms were tested on four benchmark image databases: LIVE, CSIQ, TID2013, and IVC. SROCC, PLCC, and RMSE were computed to measure the consistency and accuracy between the prediction scores and the MOS. The overall testing results are reported in Table 6. The best-performing results of FR and NR classes are highlighted in bold. 

As shown in Table 6, we can draw the following conclusions. First, compared with other state-of-the-art NR-IQA models, the proposed BM-IQE model shows competitive prediction abilities over all databases. Particularly, for the SROCC metric, BM-IQE obtains the best results on the CSIQ database and the second-best results on the LIVE, TID2013 and IVC databases, which suggests the great consistency of BM-IQE with the MOS values over various datasets. For the RMSE metric, our model outperforms all the others on each database. Second, compared with those superior FR-IQA models, BM-IQE shows a comparable quality prediction performance. Third, although the BM-IQE model didn’t obtain the best results on the IVC database, the whole result still delivers a balanced performance, which demonstrates the strong robustness of our model over all databases.

**Figure 10:**
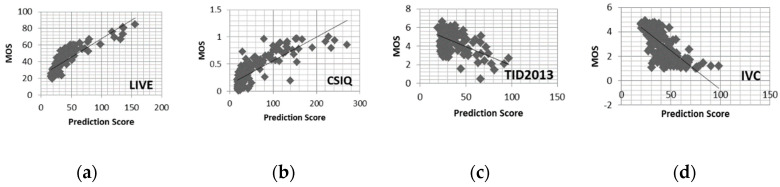
Scatter plots of objective scores predicted by BM-IQE versus subjective scores on (**a**) the LIVE image database; (**b**) the CSIQ image database; (**c**) the TID2013 image database; (**d**) the IVC image database.

Figure 10 presents the scatter plots of objective scores predicted by BM-IQE versus subjective scores (the MOS) provided by four databases. Despite the presence of some outliers, the plots are generally regression linear. In summary, when looking at the overall performance across all databases, BM-IQE demonstrates a better average performance than other NR-IQA models.

### 5.6. Performance on Particular Distortion Types

In the process of previous literature research, we found that the existing NR-IQA algorithms cannot obtain exact quality prediction results under some specific distortion types. We exam BM-IQE on five particular distortion types of the TID2013 database—non eccentricity pattern noise, Local block-wise distortions of different intensity, mean shift (intensity shift), contrast change, and change of color saturation. Four NR-IQA models were employed to compare with BM-IQE by computing the SROCC metric; they are BRISQUE, DIIVINE, NIQE, IL-NIQE, and WaDIQaM-NR. The testing results are presented in Table 7, and the best-performing results are in bold.

From Table 7, we can infer that the BM-IQE model outperforms all other algorithms under several specific distortion types. For such distortion types as non-eccentricity pattern noise, local block-wise distortions of different intensity, mean shift (intensity shift), and change of color saturation, our BM-IQE model obtains better results than the other four IQA models. Though the results are still not satisfying enough, a meaningful achievement has been made upon this. The reason for this improvement may be the adoption of texture features, which we mentioned is of great significance to visual perception in Section 5.4. However, for the contrast change distortion type, the result of BM-IQE model is barely satisfactory. One possible reason is that this distortion type is hard to characterize using existing features; another is that the color space we employed is not appropriate for the human visual perception. In future work, we need to investigate how to deal with such distortion types more properly.

### 5.7. Performance on Multiply Distorted Database

In this section, we evaluate the quality prediction performances of BM-IQE and other FR/NR-IQA algorithms on multiply distorted image databases. The LIVE MD database consists of two parts and we consider them as two separate datasets—LIVE MD1 and LIVE MD2. Images of LIVE MD1 are degraded by Gblur and JPEG distortions, and images of LIVE MD2 are degraded by Gblur and WN distortions. We compare BM-IQE with five NR-IQA models. The SROCC, PLCC and RMSE metrics were computed to evaluate the performance of each algorithm. The best-performing results are in bold, and the overall results are presented in Table 8.

As shown in Table 8, BM-IQE shows competitively performance on multiply-distorted image datasets. Specifically, compared to other algorithms, BM-IQE obtains better SROCC result which manifests better consistency with the subjective scores. Though the PLCC and RMSE values of BM-IQE are not the best-performing ones, BM-IQE shows approximate performance, which indicates that our approach designed for singly-distorted image quality assessment can also be applied to multiply-distorted cases.

### 5.8. Computational Cost

In this section, we analyze the computational cost of the proposed algorithm. All of the experiments were performed by running Matlab code on a laptop (Intel(R) Core(TM) i5-6300HQ CPU@2.3GHz, 8 GB RAM, Windows 10 Pro 64-bit, Lenovo, Beijing, China). The software platform was Matlab R2016a (MathWorks, Natick, Massachusetts, USA). The time cost of each BIQA model was measured by predicting the quality of a 512×512 color image and the results are listed in Table 9. BM-IQE has a higher computational complexity, and this may account for the procedure of block-matching, which occupies most of the running time. The computational complexity of training stage is given as O(n) and that of testing stage is given as $O\left(n^{2}\right)$.

## 6. Conclusions

In this work, we propose a BIQA model to efficaciously evaluate the quality of images for both real-life natural scenes and remote sensing scenes. Our model, BM-IQE, employs a novel block-matching strategy to help the image patch characteristics be more soundly expressed as well as adopting GLCM statistics as texture features to better describe RS images. Extensive experiments show that BM-IQE outperforms the other BIQA models on RS image datasets and achieves state-of-the-art performance on the general-purpose natural image datasets. The main achievements of our work can be summarized as following. First, BM-IQE has been confirmed suitable for the BIQA for remote sensing scenes. Therefore, it can be applied to help evaluate the imaging performance of remote sensors by assessing the visually representative RGB bands of its images. Second, for the natural image categories, the performance of the blind image quality assessment method has been meaningfully improved through the block-matching strategy.

Future work can be conducted in following dimensions. The first is the joint effect of block-matching and texture features; we can keep looking into the reasons of better prediction ability to remote sensing images. The second is that more distortion types can be adopted for investigation of RS-IQA issue, which is of great value for the subsequent remote sensing image interpretation.

## Figures and Tables

**Table 1:** Details of generated IQA database.

	Database Type	Distortion Types NO.	Reference Images NO.	Distorted Images NO.
LIVE	Single Distortion	5	29	779
CSIQ	Single Distortion	6	30	866
TID2013	Single Distortion	24	25	3000
IVC	Single Distortion	4	10	180
LIVE MD	Multiple Distortions	2	15	450

**Table 2:** Spearman Rank-Order Correlation Coefficient (SROCC) values of each feature used in BM-IQE on IQA databases.

	MSCN	MSCN Adjacent	Color	Gradient	Log-Gabor	Eng.	Ent.	Con.	Cor.
LIVE	0.8458	0.8401	0.3228	0.6393	0.8340	0.9429	0.9874	0.9786	0.9456
CSIQ	0.5262	0.4804	0.0928	0.6512	0.6418	0.9996	0.9996	0.9729	0.9183
TID2013	0.2966	0.2954	0.0416	0.3701	0.4465	0.9681	0.9834	0.9842	0.7685
IVC	0.1638	0.1566	0.0130	0.2144	0.2362	0.9631	0.9288	0.8881	0.3150

**Table 3:** Overall SROCC/Pearson linear correlation coefficient (PLCC) values of BIQA algorithm with and without block-matching.

		LIVE	TID2013	IVC	AID
Algorithm With Block-Matching	SROCC	**0.9492**	**0.8537**	**0.8703**	**0.8518**
	PLCC	**0.9020**	0.8398	**0.7922**	**0.8444**
Algorithm Without Block-Matching	SROCC	0.9354	0.8513	0.7698	0.7582
	PLCC	0.8053	**0.8651**	0.7024	0.7185

**Table 4:** Overall SROCC values of NR-IQA algorithms on remote sensing dataset.

	Distortion Types	BRISQUE	DIIVINE	NIQE	IL-NIQE	WaDIQaM	BM-IQE
The UC Merced Land Use Dataset	White Noise	0.4087	0.3068	0.3968	0.6278	0.5116	**0.6** **999**
	Gaussian Blur	0.5057	0.3599	0.4043	0.6896	0.5242	**0.7754**
	JPEG Compression	0.4232	0.3434	0.4012	0.6592	0.5876	**0.7132**
	Overall Performance	0.4459	0.3367	0.4008	0.6589	0.5411	**0.7295**
The Aerial Image Dataset	White Noise	0.5121	0.4989	0.6847	0.9235	0.9021	**0.9509**
	Gaussian Blur	0.4827	0.3863	0.5526	0.7758	0.5214	**0.7867**
	JPEG Compression	0.4179	0.3881	0.5855	0.7544	0.7357	**0.8179**
	Overall Performance	0.4709	0.4244	0.6067	0.8179	0.7197	**0.8518**

**Table 5:** Overall PLCC values of NR-IQA algorithms on remote sensing dataset.

	Distortion Types	BRISQUE	DIIVINE	NIQE	IL-NIQE	WaDIQaM	BM-IQE
The UC Merced Land Use Dataset	White Noise	0.4002	0.3113	0.3923	0.6160	0.5512	**0.6957**
	Gaussian blur	0.5021	0.3457	0.4109	0.6847	0.5197	**0.7709**
	JPEG compression	0.4077	0.3359	0.3969	0.6574	0.5844	**0.7059**
	Overall performance	0.4367	0.3310	0.4001	0.6527	0.5518	**0.7242**
The Aerial Image Dataset	White Noise	0.5445	0.4677	0.5418	0.8619	0.8896	**0.9101**
	Gaussian blur	0.4751	0.4912	0.5213	0.7510	0.5296	**0.7867**
	JPEG compression	0.5101	0.4510	0.4896	0.7286	0.6359	**0.8363**
	Overall performance	0.5099	0.4699	0.5176	0.7805	0.6850	**0.8444**

**Table 6:** Overall performance of FR/NR-IQA algorithms on LIVE, CSIQ, TID2013, and IVC databases.

	Metrics	IQA Model Type	LIVE	CSIQ	TID2013	IVC
SROCC	SSIM	FR	0.9479	0.8756	0.8075	0.9018
	FSIM	FR	**0.9634**	**0.9242**	**0.8766**	**0.9262**
	VIF	FR	0.9632	0.9196	0.7696	0.8964
	BRISQUE	NR	0.8982	0.8409	0.7166	0.6696
	DIIVINE	NR	0.8494	0.8150	0.7889	0.5266
	NIQE	NR	0.9202	0.8539	0.7887	0.8473
	IL-NIQE	NR	0.9259	0.8723	**0.8568**	**0.8776**
	WaDIQaM	NR	**0.9540**	0.7041	0.4623	0.6816
	BM-IQE	NR	0.9492	**0.8865**	0.8513	0.8703
PLCC	SSIM	FR	0.9449	0.8612	0.7736	0.9119
	FSIM	FR	0.9589	0.9091	**0.8451**	**0.9128**
	VIF	FR	**0.9598**	**0.9277**	0.8190	0.9028
	BRISQUE	NR	0.9291	0.8122	0.6259	0.6519
	DIIVINE	NR	0.8927	0.8044	0.6436	0.5973
	NIQE	NR	0.9078	0.7262	0.6264	0.8431
	IL-NIQE	NR	0.9059	0.8634	0.6411	**0.8760**
	WaDIQaM	NR	**0.9630**	0.7437	0.4641	0.6569
	BM-IQE	NR	0.9020	**0.8688**	**0.8398**	0.7922
RMSE	SSIM	FR	8.9462	0.1334	0.8511	**0.4999**
	FSIM	FR	8.3488	0.1063	**0.6595**	0.5718
	VIF	FR	**7.6676**	**0.0980**	0.7888	0.5239
	BRISQUE	NR	12.469	0.1341	0.8265	0.8192
	DIIVINE	NR	10.098	0.1130	0.5377	0.8715
	NIQE	NR	11.315	0.1344	0.7503	0.6199
	IL-NIQE	NR	11.007	0.1169	0.6044	0.5302
	WaDIQaM	NR	14.527	1.3370	1.4011	1.8538
	BM-IQE	NR	**10.032**	**0.1066**	**0.4649**	**0.4086**

**Table 7:** Overall SROCC values of NR-IQA algorithms on particular distortion types of TID2013 database.

Distortion Type	BRISQUE	DIIVINE	NIQE	IL-NIQE	WaDIQaM	BM-IQE
Non-eccentricity Pattern Noise	0.1453	0.0598	0.0698	0.0805	0.1751	**0.3865**
Local Block-wise Distortions of Different Intensity	0.2235	0.0928	0.1269	0.1357	0.3689	**0.3829**
Mean Shift (Intensity Shift)	0.1241	0.0104	0.1626	0.1845	0.1749	**0.4023**
Contrast Change	0.0403	**0.4601**	0.0180	0.0141	0.1144	0.2337
Change of Color Saturation	0.1093	0.0684	0.2460	0.1628	0.1385	**0.3748**

**Table 8:** Overall performance of NR-IQA algorithms on multiply distorted database.

	BRISQUE	DIIVINE	NIQE	IL-NIQE	WaDIQaM	MUSIQUE	BM-IQE
SROCC	0.868	0.870	0.873	0.878	0.3502	0.895	**0.907**
PLCC	0.866	0.897	0.837	0.892	0.3136	**0.908**	0.894
RMSE	8.257	8.369	10.366	8.542	12.8109	**7.930**	8.061

**Table 9:** Time cost of NR-IQA algorithms.

	Time Cost (Seconds)
BRISQUE	1.4
DIIVINE	13.2
NIQE	0.2
IL-NIQE	4.1
WaDIQaM	2.5
MUSIQUE	6.1
BM-IQE	10.9

## References

[1] Chevrel M., Courtois M., Weill G. (1981). The SPOT satellite remote sensing mission. Photogramm. Eng. Remote Sens..

[2] Karna Y.R., Hussin Y.A., Bronsveld M.C., Karky B.S. Mapping above Ground Carbon Using Worldview Satellite Image and LiDAR Data in Relationship with Tree Diversity of Forests. Proceedings of the 33rd Asian Conference on Remote Sensing.

[3] Rauchmiller R.F., Schowengerdt R.A. (1988). Measurement of Landsat Thematic Mapper Modulation TransferFunction Using an Array of Point Sources. Opt. Eng..

[4] Barry N.R., Nelson P.S. (2001). Measurement of Hyperion MTF from on-orbit scenes. Proc. SPIE Int. Soc. Opt. Eng..

[5] Leger D., Duffaut J., Robinet F. MTF Measurement Using Spotlight. Proceedings of the IGARSS ‘94–1994 IEEE International Geoscience and Remote Sensing Symposium.

[6] Wang Z., Bovik A.C., Evan B.L. Blind Measurement of Blocking Artifacts in Images. Proceedings of the 2000 International Conference on Image Processing.

[7] Liu H., Klomp N., Heynderickx I. (2010). A no-reference metric for perceived ringing artifacts in images. IEEE Trans. Circuits Syst. Video Technol..

[8] Varadarajan S., Karam L.J. An Improved Perception-Based Noreference Objective Image Sharpness Metric Using Iterative Edge Refinement. Proceedings of the 15th IEEE International Conference on Image Processing.

[9] Sheikh H.R., Bovik A.C., Cormack L. (2005). No-reference quality assessment using natural scene statistics: JPEG2000. IEEE Trans. Image Process..

[10] Moorthy A.K., Bovik A.C. (2010). A two-step framework for constructing blind image quality indices. IEEE Signal Process. Lett..

[11] Moorthy A.K., Bovik A.C. (2011). Blind image quality assessment: From natural scene statistics to perceptual quality. IEEE Trans. Image Process..

[12] Saad M.A., Bovik A.C., Charrier C. (2010). A DCT statistics-based blind image quality index. IEEE Signal Process. Lett..

[13] Saad M.A., Bovik A.C., Charrier C. (2012). Blind image quality assessment: A natural scene statistics approach in the DCT domain. IEEE Trans. Image Process..

[14] Mittal A., Moorthy A.K., Bovik A.C. (2012). No-reference image quality assessment in the spatial domain. IEEE Trans. Image Process..

[15] Ye P., Kumar J., Kang L., Doermann D. Unsupervised Feature Learning Framework for No-Reference Image Quality Assessment. Proceedings of the 2012 IEEE Conference on Computer Vision and Pattern Recognition.

[16] Zhang P., Zhou W., Wu L., Li H. SOM: Semantic Obviousness Metric for Image Quality Assessment. Proceedings of the 2015 IEEE Conference on Computer Vision and Pattern Recognition (CVPR).

[17] Zhang Y., Chandler D.M. (2013). No-reference image quality assessment based on log-derivative statistics of natural scenes. J. Electron. Imag..

[18] Zhang L., Bovik A.C. (2015). A Feature-Enriched Completely Blind Image Quality Evaluator. IEEE Trans. Image Process..

[19] Mittal A., Soundararajan R., Bovik A.C. (2013). Making a “completely blind” image quality analyzer. IEEE Signal Process. Lett..

[20] Bosse S., Maniry D., Müller K., Wiegand T., Samek W. (2018). Deep Neural Networks for No-Reference and Full-Reference Image Quality Assessment. IEEE Trans. Image Process..

[21] Zhang Y., Chandler D.M. (2018). Opinion-Unaware Blind Quality Assessment of Multiply and Singly Distorted Images via Distortion Parameter Estimation. IEEE Trans. Image Process..

[22] Long Y., Gong Y., Xiao Z., Liu Q. (2017). Accurate Object Localization in Remote Sensing Images Based on Convolutional Neural Networks. IEEE Trans. Geosci. Remote Sens..

[23] Jun L., Shen X. (2002). Image Blocking Matching Parallel Algorithms on Mean Absolute Difference. Mini Micro. Syst..

[24] Gersho A. (1982). On the Structure of Vector Quantizers. IEEE Trans. Inf. Theory.

[25] Dabov K., Foi A., Katkovnik V., Egiazarian K. (2007). Image Denoising by Sparse 3D Transform-Domain Collaborative Filtering. IEEE Trans. Image Process..

[26] Leese J.A., Novak C.S., Clark B.B. (1971). An automated technique for obtaining cloud motion from geosynchronous satellite data using cross-correlation. J. Appl. Meteorol..

[27] Alsaade F. (2012). Fast and Accurate Template Matching Algorithm Based on Image Pyramid and Sum of Absolute Difference Similarity Measure. Res. J. Inf. Technol..

[28] Barnea D.I., Silverman H.F. (1972). A Class of Algorithms for Fast Digital Image Registration. IEEE Trans. Comput..

[29] Sarwer M.G., Po L.-M., Wu Q.M.J. (2008). Fast sum of absolute transformed difference based 4×4 intra-mode decision of H.264/AVC video coding standard. Sig. Proc. Image Commun..

[30] Kunwu X., Xiaoling B., Bin A.Y. (2007). Clustering Algorithm of High-Dimensional Data Based on Units. J. Comput. Res. Dev..

[31] Zhao Y.C., Zhang C.G., Zhang S.C., Zhao L.W. (2006). Adapting K-Means Algorithm for Discovering Clusters in Subspaces. Asia-Pacific Web Conference.

[32] Wang Z., Bovik A.C., Sheikh H.R., Simoncelli E.P. (2004). Image quality assessment: From error visibility to structural similarity. IEEE Trans. Image Process..

[33] Tang H., Joshi N., Kapoor A. Learning a Blind Measure of Perceptual Image Quality. Proceedings of the IEEE Conference on Computer Vision and Pattern Recognition.

[34] Ruderman D.L. (1994). The statistics of natural images. Netw. Comput. Neural Syst..

[35] Moorthy A.K., Bovik A.C. (2009). Visual importance pooling for image quality assessment. IEEE J. Sel. Top. Signal Process..

[36] Ruderman D.L., Cronin T.W., Chiao C.-C. (1998). Statistics of cone responses to natural images: Implications for visual coding. J. Opt. Soc. Am. A.

[37] Pouli T., Cunningham D.W., Reinhard E. (2011). A survey of image statistics relevant to computer graphics. Comput. Graph. Forum.

[38] Scholte H.S., Ghebreab S., Waldorp L., Smeulders A.W.M., Lamme V.A.F. (2009). Brain responses strongly correlate with Weibull image statistics when processing natural images. J. Vis..

[39] Geusebroek J.-M., van den Boomgaard R., Smeulders A.W.M., Geerts H. (2001). Color invariance. IEEE Trans. Pattern Anal. Mach. Intell..

[40] Lasmar N.E., Stitou Y., Berthoumieu Y. Multiscale Skewed Heavy Tailed Model for Texture Analysis. Proceedings of the 2009 16th IEEE International Conference on Image Processing (ICIP).

[41] Sheikh H.R., Wang Z., Bovik A.C., Cormack L.K. Image and Video Quality Assessment Research at LIVE. http://live.ece.utexas.edu/research/quality/.

[42] Ponomarenko N., Ieremeiev O., Lukin V., Egiazarian K., Jin L., Astola J., Vozel B., Chehdi K., Carli M., Battisti F. Color Image Database TID2013: Peculiarities and Preliminary Results. Proceedings of the IEEE 2013 4th European Workshop on Visual Information Processing (EUVIP).

[43] Computational and Subjective Image Quality Lab Shizuoka University (2009). CSIQ Image Database. http://http://vision.eng.shizuoka.ac.jp/.

[44] LeCallet P., Autrusseau F. (2005). Subjective Quality Assessment IRCCyN/IVC Database. http://www.irccyn.ec-nantes.fr/ivcdb/.

[45] Jayaraman D., Mittal A., Moorthy A.K., Bovik A.C. Objective Quality Assessment of Multiply Distorted Images. Proceedings of the 2012 Conference Record of the Forty Sixth Asilomar Conference on Signals, Systems and Computers (ASILOMAR).

[46] Yang Y., Newsam S. Bag-Of-Visual-Words and Spatial Extensions for Land-Use Classification. Proceedings of the ACM SIGSPATIAL International Conference on Advances in Geographic Information Systems (ACM GIS).

[47] Xia G.-S., Hu J., Hu F., Shi B., Bai X., Zhong Y., Zhang L., Lu X. (2017). AID: A Benchmark Data Set for Performance Evaluation of Aerial Scene Classification. IEEE Trans. Geosci. Remote Sens..

[48] Zhang L., Zhang L., Mou X., Zhang D. (2011). FSIM: A feature similarity index for image quality assessment. IEEE Trans. Image Process..

[49] Sheikh H.R., Bovik A.C. (2006). Image information and visual quality. IEEE Trans. Image Process..

